# Delay in Breast Cancer: Implications for Stage at Diagnosis and Survival

**DOI:** 10.3389/fpubh.2014.00087

**Published:** 2014-07-29

**Authors:** Lee Caplan

**Affiliations:** ^1^Department of Community Health & Preventive Medicine, Morehouse School of Medicine, Atlanta, GA, USA

**Keywords:** breast neoplasms, delayed diagnosis, survival rate, patient delay, system delay

## Abstract

Breast cancer continues to be a disease with tremendous public health significance. Primary prevention of breast cancer is still not available, so efforts to promote early detection continue to be the major focus in fighting breast cancer. Since early detection is associated with decreased mortality, one would think that it is important to minimize delays in detection and diagnosis. There are two major types of delay. Patient delay is delay in seeking medical attention after self-discovering a potential breast cancer symptom. System delay is delay within the health care system in getting appointments, scheduling diagnostic tests, receiving a definitive diagnosis, and initiating therapy. Earlier studies of the consequences of delay on prognosis tended to show that increased delay is associated with more advanced stage cancers at diagnosis, thus resulting in poorer chances for survival. More recent studies have had mixed results, with some studies showing increased survival with longer delays. One hypothesis is that diagnostic difficulties could perhaps account for this survival paradox. A rapidly growing lump may suggest cancer to both doctors and patients, while a slow growing lump or other symptoms could be less obvious to them. If this is the case, then the shorter delays would be seen with the more aggressive tumors for which the prognosis is worse leading to reduced survival. It seems logical that a tumor that is more advanced at diagnosis would lead to shorter survival but the several counter-intuitive studies in this review show that it is dangerous to make assumptions.

## Introduction

Breast cancer continues to be a disease with tremendous public health significance. It is estimated that in the United States in 2014 approximately 232,670 cases of female breast cancer will be diagnosed, and approximately 40,000 women will die from the disease, making breast cancer the second leading cause of cancer deaths in women (1). Primary prevention of breast cancer is still not available except by extreme measures such as prophylactic mastectomy for women who are genetically at high risk, so efforts to promote early detection continue to be the major focus in fighting breast cancer. The goal of early detection is to diagnose and treat breast cancer patients in an early stage when the prognosis for long-term survival is best. Prognosis is generally more favorable for women with early stage disease than for those with more advanced disease. Since early detection is associated with decreased mortality, one would think that it is important to minimize delays in detection, diagnosis, and treatment. Longer waiting times prior to breast cancer diagnosis and the initiation of therapy are of prognostic concern if delay leads to stage progression, disease worsening, or treatment complications. There are two major types of delay. Patient delay is delay in seeking medical attention after self-discovering a potential breast cancer symptom or failure to keep appointments. System delay is delay within the health care system in getting appointments, scheduling diagnostic tests, receiving a definitive diagnosis, and initiating therapy. Both of these, by leading to delays in diagnosis and treatment, could result in a poorer prognosis for women with breast cancer.

A comprehensive review of the literature on delay in breast cancer done in the early 90s consisted of several components (2). The first part of the review examined studies that explored the relationship between delay and prognosis. The review then shifted to the importance of patient delay and the factors that are associated with long patient delay. It did the same thing with system delay. Since that time, a number of review articles have been published exploring the factors associated with long delay (3–5). The author therefore decided to focus this review on the impact of delay on survival from breast cancer.

## Early Studies

Earlier studies of the consequences of delay on prognosis tended to show that increased delay is associated with more advanced stage cancers at diagnosis, thus resulting in poorer chances for survival. In fact, a meta-analysis of 87 studies suggested strongly that women who begin treatment 3–6 months after the appearance of breast cancer-related symptoms have significantly worse survival than women who wait <3 months (6). However, most of those studies were old, with a majority taking place prior to 1970. Two studies that took place in the late 1990s that were included in the review are discussed below among the recent studies (7, 8).

A number of additional studies from the 80s that were not included in the review by Richards et al. also showed that increased delay was associated with more advanced tumors at diagnosis and/or reduced survival. Robinson et al. looked at delay in diagnosis among breast cancer patients and found that those with delays of at least 6 weeks had more advanced disease (35% stage I, 52% stage II, and 12% stage III) than those with delays of <6 weeks (52% stage I, 42% stage II, and 5% stage III) (9). Survival was not determined in this study. Neale et al. demonstrated that even without adjustment for tumor grade, patients at Houston’s M.D. Anderson Hospital with delay of more than 6 months had substantially lower cumulative survival after 10 years than patients with delay of 3–6 or <3 months (10). In a study of breast cancer patients at New York’s Memorial Sloan-Kettering Cancer Center, Pilipshen et al. reported that among patients treated by radical or modified radical mastectomy in 1970–1971 and 1980–1981 combined, those delaying more than 6 months were approximately twice as likely to have tumors of at least 4 cm and approximately 40% more likely to have axillary metastases than patients delaying ≤6 months (11). In a study of 274 Los Angeles breast cancer patients, increased relative risks for regional disease were observed in the interval between self-discovery of the symptom and first contact with the physician up to 5 months (12). In addition, regional disease was associated with the interval between self-discovery of the symptom and biopsy, but not with the interval between the first contact with the physician and biopsy. The authors found that once the patient was seen by the doctor, there was no evidence that further delays adversely affected the likelihood of being diagnosed with regional disease.

It is important to note that several very early studies from the 1940s to 1950s had not detected the expected inverse relationship between delay and survival (13, 14). In response, Bloom showed that this was largely due to confounding by histologic grade or the inherent malignant potential of the cancers (15). In an analysis of breast cancer cases from the Middlesex Hospital in London, it appeared that women with long delay were more likely to have less aggressive cancers.

## Recent Studies

Among the more recent studies, the results are mixed. One group of studies analyzes the relationship between delay and tumor stage at the time of initiation of therapy. One may infer that women with more advanced tumors will, on average, have shorter survival. Other studies analyze the relationship between delay and survival time, sometimes without considering tumor stage. These two types of studies are reviewed separately.

## Studies of Delay and Tumor Stage

Plotogea et al. performed two studies in which longer delay was found to be associated with lower stage at diagnosis. In one of the studies, out of a random sample of 2,615 women aged 50–69 with invasive breast cancer diagnosed between January 1, 1995 and December 31, 2003, 2,065 were classified into three groups: *screen-detected breast cancer*, if the patient had a mammogram during the study period and her breast cancer was diagnosed within 6 months of that screen; *interval breast cancer*, if the cancer occurred within 6–12 months of a mammogram; and *symptomatic breast cancer*, if the cancer was detected in a woman who did not have a screening mammogram during the study period prior to diagnosis (16). Screen-detected breast cancers diagnosed at stage II and symptomatic cancers diagnosed at stage III or IV had significantly shorter diagnostic wait times (time from imaging or biopsy to breast cancer diagnosis) compared to those diagnosed at stage I (OR = 0.66, 95% CI = 0.50–0.87 and OR = 0.46, 95% CI = 0.25–0.85, respectively). Plotogea’s other study included 1,760 women aged 50–69 diagnosed in Ontario with invasive breast cancer from 1995 to 2003 (17). The median delays were 17 days from diagnosis to definitive surgery, 44 days from final surgery to postoperative chemotherapy, and 75 days from final surgery to postoperative radiotherapy. Of note is the finding that diagnosis during 2000–2003 was associated with significantly longer delay for each phase of the treatment pathway compared to 1995–1999. Higher stage at diagnosis was associated with shorter wait times from diagnosis to definitive surgery (stage III versus I: OR = 0.49, 95% CI = 0.34–0.71).

Warner et al. conducted a study on 21,427 White, Hispanic, Black, and Asian women diagnosed with stage I–IV breast cancer between January 1, 2000 and December 31, 2007 at a National Comprehensive Cancer Network Center (18). Among the symptomatic women, median time to diagnosis ranged from 36 days in Whites to 53.6 days in Blacks, while among women with abnormal mammograms, median time to diagnosis ranged from 21 days in Whites to 29 days for Blacks. Blacks had the highest proportion of stage III or IV tumors (26%). After accounting for time to diagnosis, the observed increased risk of stage III and IV breast cancer was reduced from 40 to 28% among Hispanics and from 113 to 100% among Blacks, but estimates remained statistically significant. Longer time to diagnosis did not completely explain the observed differences in stage between racial/ethnicity groups.

Two hundred Libyan women, aged 22–75 years, with breast cancer diagnosed during 2008–2009 were interviewed about their diagnosis delay [interval from the date of the first symptoms to the date of final breast cancer diagnosis based on histopathological examination (19)]. The median diagnosis delay was 7.5 months, as 30.0% of patients were diagnosed within 3 months after symptoms, 14% were diagnosed within 3–6 months, and 56% within a period longer than 6 months. Diagnosis delay of >3 months was associated with bigger tumor size, positive lymph nodes, high incidence of late clinical stages, and metastatic disease.

A study examined the relationships between religiosity, spirituality, breast cancer fatalism, disclosure of symptoms, marital status, and time to seek medical care and breast cancer stage in African-American women with self-detected breast changes (20). A convenience sample of 129 African-American women aged 30–84 who self-reported detecting a breast symptom before diagnosis of breast cancer within the preceding 12 months were included in the study. Most women delayed more than 3 months in seeking medical care. No associations were found between religiosity, spirituality, and fatalism and time to seek medical care. Women who delayed seeking medical care for longer than 3 months were more likely to present with a later stage of breast cancer than women who sought care within 3 months of discovering their symptom. A significant association was found between time to seek medical care and breast cancer stage at diagnosis, as the longer the delay the more advanced was the breast cancer stage (*P* = 0.01). Logistic regression showed a significant positive association between delay of symptom presentation for medical diagnosis and stage of breast cancer (OR = 6.37, 95% CI = 2.84–14.30).

Redondo et al. performed a study of 411 breast cancer patients who were histologically diagnosed at the Costa del Sol Hospital, Marbella, Spain between January 1, 1996 and December 31, 2001 to evaluate the influence of emergency admission and delays on prognosis (21). Total delay was divided into three components. Patient and primary care delay was defined as the interval from first symptom to first visit to hospital; diagnostic delay as the interval from first hospital visit to definitive histopathologic diagnosis, and treatment delay as the interval from diagnosis to first treatment. The median delay times were 87, 11, and 36 days, respectively. Patient and primary care delay of >6 months was associated with advanced stages, although these tumors presented pathologic characteristics of good prognosis, such as positive estrogen and progesterone-receptor status and non-ductal type. In contrast, a diagnostic delay of <30 days was significantly associated with increased tumor size, lymph node metastasis, and ductal type (*P* < 0.05), and with a negative progesterone-receptor status and poorly differentiated tumors (*P* < 0.01). The authors attributed this to sicker patients receiving prompt medical attention. Treatment delay of <30 days was significantly associated with ductal type, negative hormone receptor status, and the presence of signs or symptoms other than a lump (*P* < 0.05).

Elzawawy et al. performed several studies between 1984 and 2007 looking at the delay in seeking medical advice and the pathological tumor size of female breast cancer patients in Port Said, Egypt (22). Over the years, there was a decline in delay, and there was also a decline in advanced cases.

Elmore et al. conducted a retrospective cohort study on 400 female breast cancer patients diagnosed between 1985 and 1993 and followed through June 20, 2001 (23). Among the 117 women with patient-detected breast cancer, those who completed their evaluation within 30 days were significantly more likely to have local disease at diagnosis (83%) than women taking more than 30 days to have an evaluation who were more likely to be diagnosed with regional disease (36%, *P* < 0.05). Women taking >30 days for an evaluation were significantly more likely to have a breast cancer recurrence or death than women who had an evaluation in <30 days (24 versus 8%; *P* < 0.05).

Patient delay (time from onset of first symptoms to first consultation of a doctor) and its impact on stage of disease were examined in a population-based study of 287 women aged 18–80 with newly diagnosed invasive symptomatic breast cancer (24). Median patient delay was 16 days among symptomatic patients, and 18% of all breast cancer patients had patient delay longer than 3 months. The results demonstrated a difference in the association between patient delay and stage at diagnosis of breast cancer when stratified by tumor differentiation. For women with well differentiated tumors, the proportion of late-stage breast cancer did not change with increasing patient delay (*P*_trend_ = 0.83), but for women with poorly differentiated tumors, a monotonic trend between length of patient delay and late-stage diagnosis was observed (*P*_trend_ = 0.03).

In a study to examine the extent and determinants of patient and general practitioner delay in the presentation of breast cancer, 185 cancer patients attending a breast unit were interviewed 2 months after diagnosis (8). Patient delay in presentation to the general practitioner was 12 or more weeks in 19% of the patients and was related to clinical tumor size ≥4 cm (*P* = 0.0002) and with a higher incidence of locally advanced and metastatic disease (*P* = 0.01).

Lannin et al. conducted a case–control study of patients diagnosed as having breast cancer at the University Medical Center of Eastern Carolina from 1985 through 1992 (25). There were a total of 540 patients and 414 matched controls from the community. It was found that delaying seeing a physician because of money was a predictor of advanced breast cancer stage (OR = 1.6, 95% CI = 1.1–2.5).

## Studies of Delay and Survival

Treatment delay (the number of weeks between the date of diagnosis and date of definitive treatment) was evaluated in a retrospective case-only study on 8,860 adolescent and young adult breast cancer cases diagnosed from 1997 to 2006 using the California Cancer Registry database (26). The 5-year survival in women who were treated by surgery and had treatment delay of more than 6 weeks was 80% compared with 90% (*P* = 0.005) in those with treatment delay of <2 weeks. In multivariate analysis, longer treatment delay was a significant risk factor for shorter survival. The adverse effect of increased treatment delay on survival was more pronounced in African-American women, those with public or no insurance, and those with low socioeconomic status.

McLaughlin et al. conducted a retrospective analysis of 1,786 low-income, adult female North Carolina Medicaid enrollees diagnosed with breast cancer from January 1, 2000 to December 31, 2002, in the linked North Carolina Central Cancer Registry-Medicaid Claims database to study the impact of long delay between a biopsy-confirmed breast cancer diagnosis and treatment initiation on survival (27). The median delay was 22 days. Adjusted Cox proportional hazards regression demonstrated that the delay interval did not affect survival among those diagnosed at an early stage, but among late-stage patients, intervals between diagnosis and first treatment ≥60 days were associated with significantly worse breast cancer-specific survival (HR = 1.85, 95% CI = 1.04–3.27; *P* = 0.04).

In a study of 553 patients in Pittsburgh with breast cancer metastasis, treatment delay was defined as the time in days between the date of diagnosis of initial breast cancer metastasis (the date of first metastatic biopsy or CT scan) and the date of the initiation of first treatment (28). Treatment delays of over 12 weeks were related to adverse survival outcomes.

Brazda et al. conducted a retrospective review of patients undergoing breast cancer treatment between August 2005 and December 2008 in a comprehensive, multidisciplinary breast oncology program in two hospital systems (29). The patients were divided into three groups based on interval to treatment (the time between date of pathological diagnosis, usually via core needle biopsy, and the date of initial therapy, either surgical or systemic): 0–45, 46–90, and >90 days, and there was no association between the interval to treatment and survival. Because previous studies had revealed decreased survival with delays >90 days, the authors separated patients into groups based on interval to treatment of <90 and >90 days, but there was no demonstrable difference in survival between these two groups.

Smith et al. conducted a study of 314 participants in South Carolina’s Breast and Cervical Early Detection Program between 1996 and 2004 to assess differences in factors associated with breast cancer mortality in African-American as compared to European American women (30). The study looked at three delay intervals, diagnostic delay, treatment delay, and total delay. The diagnostic delay was the time interval between the date of the first screening examination (clinical breast exam or mammography) that found an abnormality and the date of the pathologic diagnosis of breast cancer within that screening cycle. Treatment delay was the time interval between the date of the pathologic diagnosis of breast cancer and the date of the initiation of the first course of treatment. There was no significant association between the risk of death and the delay intervals using Cox Proportional Hazards models.

Hershman et al. conducted a study in women in the Henry Ford Health System tumor registry who were diagnosed with stage I/II breast cancer between January 1, 1996 and December 31, 2001 and who received adjuvant chemotherapy (31). An observed/expected ratio of treatment duration and completed chemotherapy cycles was calculated for each patient. Among the 344 patients receiving the expected number of cycles, 60% experienced delays, but these delays did not reduce survival. However, the authors cautioned that only 17% of the patients had delays longer than 2 weeks, so perhaps the delays may not have been long enough to affect survival or perhaps the study may have been underpowered to detect their effects.

Sainsbury et al. conducted a retrospective analysis of breast cancer patients listed in the Yorkshire Cancer Registry to investigate whether delays by providers in routine practice for diagnosis influence survival (32). Information for 36,222 patients with breast cancer from 1976 to 1995 was available. Patients were grouped according to the time from family physician referral to treatment, with the times being <30, 30–59, 60–89, and 90 days or more. Median delay between first hospital visit and treatment nearly doubled from 7 to 13 days between 1976 and 1995, while the median delay from family physician referral to hospital visit changed only from 10 to 12 days in the same time period. Patients with delays <30 days between family physician referral and treatment had worse survival than patients with the longer delay time periods (*P* < 0.001).

In a study of 7,608 patients with primary breast cancer, patient’s and doctor’s delays were arbitrarily divided into short (0–14 days), intermediate (15–60 days), and long (>60 days) (33). A long patient’s delay was associated with an unfavorable outcome when compared with a short patient’s delay, but the opposite was true for doctor’s delay as the prognosis was better for patients with long doctor’s delay compared to those with short doctor’s delay. In addition, when corrected for age, the prognostic value of delay on mortality increased by 24% for a long patient’s delay compared to a shorter one and by 13% for a short doctor’s delay compared to a longer one. The authors suggested that the doctor’s delay findings indicate that doctors are capable of distinguishing between more and less aggressive malignancies.

Richards et al. conducted a study of 2,964 women who presented with any stage of breast cancer to Guy’s Hospital in London between 1975 and 1990 (7). A total of 32% of the women had symptoms for at least 12 weeks prior to their first hospital visit. Among these women, 32% had locally advanced or metastatic disease, compared to 10% of those women with delays of <12 weeks. Women with delays of 12–26 weeks had significantly worse survival rates than those with <12 weeks of delay. In multivariate analyses, the adverse impact of delay on survival was attributable to an association between longer delays and more advanced stage, but this adverse impact disappeared within individual stages of disease.

## Conclusion

Despite the prevalence of studies suggesting that long delays are associated with poorer survival, given the mixed results for the more recent studies, it is hard to make a case one way or another for longer delay leading to reduced survival. It seems counter-intuitive that longer delays should be associated with earlier stage cancers and/or increase survival compared to shorter delays. However, there are a number of possible explanations for this phenomenon. One explanation is that diagnostic difficulties could perhaps account for this survival paradox (34). A rapidly growing lump may suggest cancer to both doctors and patients, while a slow growing lump or other symptom could be less obvious to them. If that were the case, then one would expect to see less delay in the woman with the symptom that is more suggestive of cancer. So the biological characteristics of the tumor might be the driver of the poorer survival, as opposed to the short delay. Another possible explanation could be that a study did not have enough “long” delayers to have adequate power. A suggestion to that effect was made by Hershman et al. regarding their study. Finally, it is possible that the delay intervals that were used in some studies to categorize “long” delayers were just not long enough to have a negative effect on survival. That coupled with one or both of the other explanations could help explain how some studies showed a protective effect of delay.

One notes that there are more studies of delay as it relates to tumor stage or size than there are of studies that focus on delay as it relates to survival. This may be explained by the greater difficulty of conducting studies of survival. Tumor staging occurs early in the course of treatment, while survival cannot be determined until the death of the patient. However, studies of survival probably deserve greater credence. It seems logical that a tumor that is more advanced at diagnosis would lead to shorter survival; but the several counter-intuitive studies in this review show that it is dangerous to make assumptions.

## Conflict of Interest Statement

The author declares that the research was conducted in the absence of any commercial or financial relationships that could be construed as a potential conflict of interest.
